# pRb Inactivation in Mammary Cells Reveals Common Mechanisms for Tumor Initiation and Progression in Divergent Epithelia

**DOI:** 10.1371/journal.pbio.0020022

**Published:** 2004-02-17

**Authors:** Karl Simin, Hua Wu, Lucy Lu, Dan Pinkel, Donna Albertson, Robert D Cardiff, Terry Van Dyke

**Affiliations:** **1**Department of Genetics, Lineberger Comprehensive Cancer CenterThe University of North Carolina School of Medicine, Chapel Hill, North CarolinaUnited States of America; **2**Comprehensive Cancer Center, University of CaliforniaSan Francisco, San Francisco, CaliforniaUnited States of America; **3**Center for Comparative Medicine, University of CaliforniaDavis, Davis, CaliforniaUnited States of America

## Abstract

Retinoblastoma 1 (pRb) and the related pocket proteins, retinoblastoma-like 1 (p107) and retinoblastoma-like 2 (p130) (pRb_f_, collectively), play a pivotal role in regulating eukaryotic cell cycle progression, apoptosis, and terminal differentiation. While aberrations in the pRb-signaling pathway are common in human cancers, the consequence of pRb_f_ loss in the mammary gland has not been directly assayed in vivo. We reported previously that inactivating these critical cell cycle regulators in divergent cell types, either brain epithelium or astrocytes, abrogates the cell cycle restriction point, leading to increased cell proliferation and apoptosis, and predisposing to cancer. Here we report that mouse mammary epithelium is similar in its requirements for pRb_f_ function; Rb_f_ inactivation by T_121_, a fragment of SV40 T antigen that binds to and inactivates pRb_f_ proteins, increases proliferation and apoptosis. Mammary adenocarcinomas form within 16 mo. Most apoptosis is regulated by p53, which has no impact on proliferation, and heterozygosity for a *p53* null allele significantly shortens tumor latency. Most tumors in *p53* heterozygous mice undergo loss of the wild-type *p53* allele. We show that the mechanism of *p53* loss of heterozygosity is not simply the consequence of Chromosome 11 aneuploidy and further that chromosomal instability subsequent to *p53* loss is minimal. The mechanisms for pRb and p53 tumor suppression in the epithelia of two distinct tissues, mammary gland and brain, are indistinguishable. Further, this study has produced a highly penetrant breast cancer model based on aberrations commonly observed in the human disease.

## Abstract 2

Inactivation of the three retinoblastoma genes in the mouse mammary gland provides a new animal model for human breast cancer

## Introduction

Aberrant retinoblastoma 1 (pRb) pathway activity, resulting from defects in pRb itself, cyclin-dependent kinase inhibitor 2A (p16^INK4a^), cyclin D1 (CCND1), or cyclin-dependent kinase 4 (CDK4), is observed in the majority of human sporadic cancers (Marshall 1991; Weinberg 1995; Sherr 1996; Ortega et al. 2002). This pathway is commonly altered early in cancer development, indicating an ability to predispose cells to tumorigenesis. However, whether the mechanism(s) is similar among cell types is not known. Examination of pRb inactivation in specific cell types in vivo has been technically challenging due to the apparent functional compensation or redundancy among pRb, retinoblastoma-like 1 (p107), and retinoblastoma-like 2 (p130) in many cell types of the mouse (Luo et al. 1998; Robanus-Maandag et al. 1998; Dannenberg et al. 2000; Sage et al. 2000). Thus, genetic inactivation of the *Rb* gene alone, either by conditional deletion (Marino et al. 2000) or by the generation of chimeric mice harboring pRb-deficient cells (Maandag et al. 1994; Williams et al. 1994) yields only medulloblastomas, pituitary, and thyroid tumors.

We have begun to systematically examine the role of retinoblastoma protein family (pRb_f_) inactivation in multiple cell types of the mouse by dominant expression of T_121_, a truncation mutant of simian virus 40 (SV40) T antigen that inactivates all three pRb-related proteins (DeCaprio et al. 1989; Dyson et al. 1989; Ewen et al. 1989; Stubdal et al. 1997; Sullivan et al. 2000). In this report we determine the role of pRb inactivation in mammary adenocarcinoma predisposition, establish a role for p53 inactivation in subsequent mammary adenocarcinoma progression, and, together with our previous studies, provide a comprehensive comparison of these mechanisms in distinct epithelial lineages.

pRb plays a critical role in eukaryotic cell cycle progression, when cells exit G0 or G1 and enter S phase, thereby acting as a crucial negative regulator of cellular proliferation and neoplasia (Sherr and McCormick 2002). In quiescent or early G1-phase cells, pRb is hypophosphorylated and associates with specific members of the E2F transcription factor family, converting them to active transcriptional repressors (Hamel et al. 1992; Weintraub et al. 1992). Gene repression is also mediated by pRb and p130 recruitment of histone deacetylase to promote formation of inhibitory nucleosomes (Brehm et al. 1998; Luo et al. 1998; Magnaghi-Jaulin et al. 1998). The many proteins found in association with pRb suggest other regulatory mechanisms may also be involved (Morris and Dyson 2001), although the biological potential for most of these interactions remains yet unproven. Cell cycle progression from G to S phase occurs when complexes of D-type cyclins/CDK4/CDK6 phosphorylate pRb, thereby derepressing E2Fs to direct transcription of DNA-replication machinery and nucleotide biosynthesis genes (Dyson 1998).

Like most human solid tumors, breast cancers harbor frequent alterations in the pRb pathway, including CCND1 overexpression in 45% (Buckley et al. 1993), p16^INK4A^ loss in 49% (Geradts and Wilson 1996), and pRb loss in 6% of breast tumors (Geradts and Wilson 1996). In the *Rb*-deficient mouse mammary gland, p107 and/or p130 may play overlapping or compensatory roles, as they do during embryonic development, given that pRb is dispensable for normal mammary development and mammary tumor suppression. pRb-deficient embryonic stem cells participate in normal mammary gland formation in chimeric mice (Maandag et al. 1994), and donor *pRb^−/−^* mammary precursor cells transplanted into wild-type mice can populate a normal mammary gland without evidence of neoplasia, even after multiple pregnancies (Robinson et al. 2001).

The interplay between pRb signaling and the tumor protein p53 pathway is also critical to the understanding of breast cancer biology. Since the pRb pathway is defective in a majority of human tumors and the *p53* gene is mutated in about half of them, including approximately a fifth of sporadic breast cancers (Nigro et al. 1989; Greenblatt et al. 1994), these aberrations often coexist. Whether loss of these tumor suppressor pathways collaborate in tumorigenesis is also cell type-specific. In a brain epithelial tumor model, we previously demonstrated that, in the absence of pRb_f_ function, inactivation of p53 significantly decreases apoptosis and accelerates tumor growth in vivo (Symonds et al. 1994). However, in astrocytic brain tumors induced by pRb_f_ inactivation, tumor progression is not accelerated by reduced p53 activity; rather, the phosphatase and tensin homolog (*PTEN*) regulates the apoptosis, and reduction in its function accelerates tumor growth (Xiao et al. 2002).

In this report, we extend our analysis of pRb function in vivo and examine the consequence of pRb_f_ loss specifically in mammary epithelium. These studies serve not only to provide insight into the cell specificity of tumor suppression mechanisms, but also to model the stepwise evolution of breast adenocarcinomas that harbor defects in this pathway.

## Results

### Generation of Mice with Inducible pRb_f_ Deficiency in Mammary Cells

**Figure 1:**
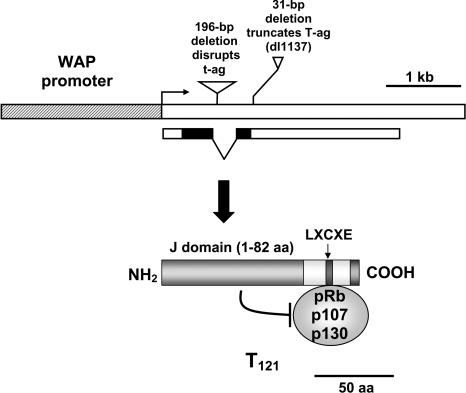
Diagram of the *WAP-T_121_* Transgene and Protein The fragment consists of the 2.4 kb WAP promoter (hatched) and the mutant SV40 T-antigen coding region (white box) containing two deletions, the 196-bp amino-terminal deletion, which abolishes small t antigen production, and the *dl*1137 deletion, which truncates T antigen. Both the J domain and the LXCXE domain are required for pRb family inactivation (see Materials and Methods).

**Figure 2:**
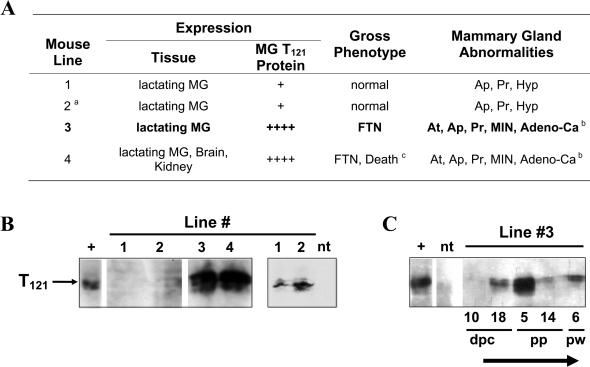
Expression of T_121_ Protein in *WAP-T_121_* Mice and a Summary of Gross Phenotypes As expected, each line showed mammary-specific expression following lactation induction, while line 4 showed more widespread expression, with protein detected in brain and kidney. Mice from the higher-expressing lines 3 and 4 failed to nurse because of lactation defects. Mammary glands of adult female mice from all four lines showed elevated proliferation and apoptosis. Glands from line 1 and 2 mice were hyperplastic, while glands from lines 3 and 4 were atrophic. Lines 3 and 4 later developed carcinomas and other neoplasms. T_121_ protein was detected by Western blot analysis in lactating mammary glands of animals from all four lines (B), although the lower-expressing lines 1 and 2 required immunoprecipitation with anti-T-antigen antibody prior to Western blot analysis (right panel in [B]). Brain tumor extract (see Materials and Methods) was used for a positive control, and nontransgenic mammary tissue extract was used for a negative control. A timecourse analysis of T_121_ expression (C) shows lactation-induced expression peaking at 5 d postpartum. **Abbreviations:** Adeno-Ca, adenocarcinoma; AP, elevated apoptosis in mammary gland; At, atrophy; dpc, postcoital; FTN, failure to nurse; Hyp, hyperplastic acini; MG, mammary gland; MIN, mammary epithelia neoplasia; ND, not determined; nt, nontransgenic; pp, postpartum; Pr, elevated proliferation in mammary gland; pw, post-weaning. ** Footnotes:** ^a^Mosaic founder animal.^b^At earlier stages, development defects attributed to atrophy, while MIN and adenocarcinoma were observed at terminal stages.^c^Approximately half of progeny died of unknown cause.

Seven founder mice were generated in which the *T_121_* gene was regulated by the whey acidic protein (WAP) transcriptional signals (Figure 1; see Materials and Methods). Of these, two founder animals died spontaneously of unknown causes, while the transgenic progeny of the third line died prematurely, also of unknown cause (Figure 2A). The extent to which the transgene contributed to these deaths was not investigated further; however, ectopic transgene expression was detected in several tissues (data not shown). Characterization of female mice of the four remaining lines is the focus of this report.

### T_121_ Is Expressed in Lactating Mammary

Western immunoblotting analyses of mammary gland extracts demonstrated that this tissue expresses T_121_ protein at the expected size in all four lines (Figure 2B). T_121_ expression in lines 1 and 2 was only revealed following immunoprecipitation using an anti-T-antigen antibody prior to Western blot analysis, indicating lower levels of T_121_ (right panel in Figure 2B). A survey of select tissues showed that detectable expression was restricted to the mammary gland in lines 1–3, while expression was more widespread in the higher expressing line 4 (data not shown) and included brain and kidney expression. As expected, T_121_ expression was induced by lactation with highest levels observed 5 d postpartum (Figure 2C). Southern blot analyses indicate that mice in line 3, which was used as a representative line for extensive characterization, harbor approximately ten copies of the transgene at a single insertion site (data not shown).

### Impact of Rb_f _Inactivation in Mammary Epithelium 

**Figure 3:**
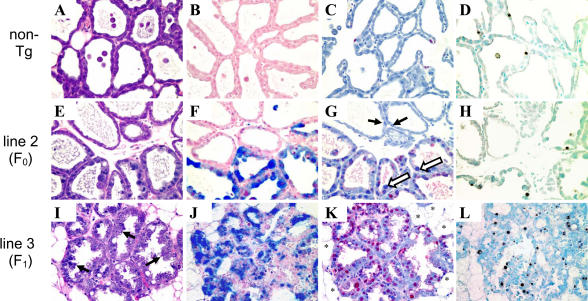
Mammary-Specific Inactivation of the pRb Pathway Induces Extensive Abnormalities Histologic comparisons of nontransgenic (A–D), mosaic (F_0_ line 2 [E–H]), and transgenic (F_1_, line 3 [I–L]) lactating mammary glands reveals that T_121_ expression results in increased proliferation and apoptosis. Hemotoxylin and eosin staining shows acini of the normal lactating gland are composed of a single layer of secretory epithelial cells (A) with milk-filled lumen. Consistent with atrophy, transgenic animals have a lower density of acini demonstrated by the presence of lipid-filled adipocytes (asterisk in [K]). Acini composed of T_121_-expressing cells are atypical. Many are collapsed and composed of tall columnar epithelia of large hyperchromatic cells with papillary tufting (arrows in [I]). Transgene-expressing cells have large pleomorphic nuclei (open arrows in [G]) as compared to nuclei of nonexpressing cells (arrows in [G]). Staining for T_121_ expression (blue in [B]–[J]) indicates the line 2 F_0_ animal is mosaic, showing localized expression (F), whereas the transgene expresses throughout the gland of an F_1_ line 3 animal (J). Increased proliferation assayed by PCNA staining (red) is also localized in the mosaic founder (G), but found throughout the F_1_ transgenic gland (K). Similarly, TUNEL staining (brown) demonstrates increased apoptosis in transgenic animals (H and L); moreover, the regionalized apoptosis in the mosaic gland (H) strongly suggests that transgene expression and not precocious involution is the cause. All samples are from primiparous females on lactation day 1.

**Figure 4:**
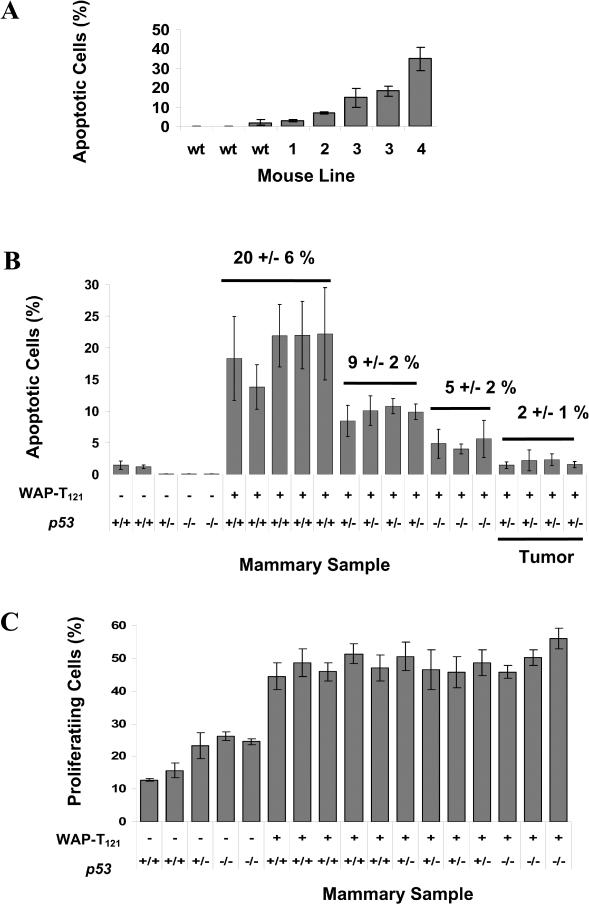
Reduced p53 Activity Decreases Apoptosis but Does Not Increase Proliferation Representative apoptosis levels of each mouse line correlate with T_121_ expression as indicated by the percentage of TUNEL positive cells (A). Decreasing levels of p53 activity correlate with lower levels of apoptosis in transgenic mammary glands (B). The mean percentage of apoptotic cells in *p53* wild-type transgenic glands was 21%; in *p53* heterozygous animals, 9%; and in *p53* null animals, 5% (B), indicating that 75% of the apoptosis is p53-dependent. Apoptosis levels are further reduced to 2% in terminal stage tumors (B, Tumors). The percentage of PCNA staining cells remains unchanged in *p53* heterozygous or nullizygous animals (C), indicating that reduction of p53 activity levels had no significant impact on cell proliferation. Samples were derived from primiparous animals on lactation day 1, except as indicated as tumor samples (B). Transgenic animals in (B) and (C) were from line 3.

Representative histological analysis of lactating mammary glands (day 1) from single-pregnancy females of the line 2 founder (F_0_) and a line 3 F_1_ mouse shows that the impact of *Rb* perturbation is severalfold. Compared to an age- and parity-matched control tissue, the normal architecture of the lactating mammary tissue is disturbed. In contrast to normal tissue where acini consist of a single layer of secretory epithelia with milk-filled lumen (Figure 3A), transgenic animals have a lower density of acini (Figure 3K), consistent with atrophy, and are often atypical (Figure 3I). T_121_-positive mammary epithelial cells were associated with abnormalities (Figure 3B, 3F, and 3J). The line 2 F_0_ animal was mosaic for T_121_ protein expression with distinct regions of expressing and nonexpressing cells (Figure 3F), whereas T_121_ expression in the line 3 animal was in secretory epithelium distributed throughout the gland (Figure 3J). Increased proliferation, indicated by proliferating cell nuclear antigen (PCNA) staining, was also observed in transgenic mammary glands (Figure 3C, 3G, and 3K), concomitant with increased levels of apoptosis assayed by TUNEL staining (Figure 3D, 3H, and 3L). Quantification of T_121_ expression and apoptosis revealed higher protein expression levels (see Figure 2B) correlate with higher percentages of apoptotic cells (Figure 4A). Consistent with a model for cell-autonomous functioning of T_121_, the pattern of abnormalities of morphology, proliferation, and apoptosis in the mosaic animal mimicked the regionalized T_121_ expression pattern, and conversely, where T_121_ protein was absent, the tissue appeared normal.

### Role of p53 in Apoptosis

To investigate the impact of germline loss of p53 on apoptosis levels in Rb_f_-deficient mammary glands, we mated line 3 animals to *p53* null mice to generate transgenic and nontransgenic females of distinct *p53* genotypes (+/+, +/−, −/−). Transgene expression was induced by a single pregnancy, and mammary glands were examined on lactation day 1. As expected, nontransgenic mammary glands showed no appreciable apoptosis regardless of *p53* status (Figure 4B). However, in transgenic animals, decreased levels of p53 activity were correlated with lower levels of apoptosis. The mean percentage of apoptotic cells in *p53* wild-type transgenic glands was 21%; in *p53* heterozygous animals, 9%;and in *p53* null animals, 5% (Figure 4B), indicating that 75% of the apoptosis is p53-dependent. That we could detect haploinsufficiency of *p53* for apoptosis is remarkable, since in the previously characterized T_121_-expressing choroid plexus epithelium, apoptosis levels were the same in *p53* heterozygous and wild-type backgrounds (Lu et al. 2001). This observation indicates that there is a threshold for p53 levels in eliciting apoptosis and that either the threshold is different between cell types or that the absolute functional p53 level is distinct. Such differences could have significant impact on the requirements for tumorigenesis.

### Role of p53 in Proliferation

In two other transgenic mouse models of breast cancer, where tumors were initiated by activated Harvey rat sarcoma viral oncogene homolog (v*-Ha-ras*) (Hundley et al. 1997) or *wingless*-related murine mammary tumor virus (MMTV) integration site 1 (*Wnt-1*) (Donehower et al. 1995), inactivation of p53 did not result in a reduction of apoptosis; rather, loss of p53 was associated with increased proliferation of the mammary epithelium. To determine whether p53 inactivation also impacted mammary cell proliferation induced by Rb_f_ inactivation, glands from primiparous lactating (day 1) mice were assessed for the expression of nuclear PCNA. Unlike the tumors initiated by activated *Ras* or *Wnt-1*, *p53* heterozygosity or nullizygosity had no significant impact on the level of cell proliferation (Figure 4C). This experiment indicates that p53 can have distinct mechanisms of action depending on the nature of the initiating lesion.

### pRb Inactivation Predisposes to Tumorigenesis

**Figure 5:**
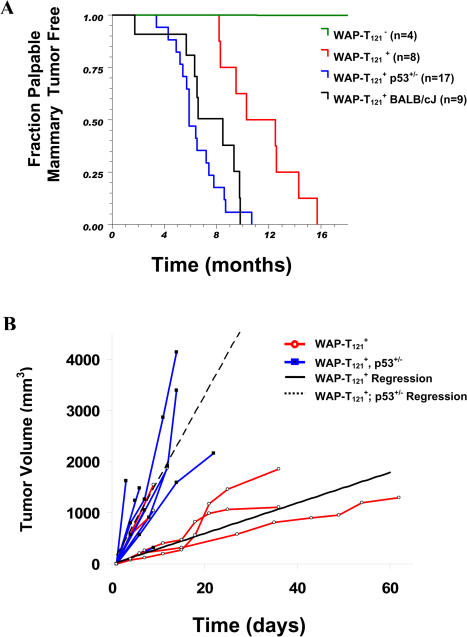
Mammary Tumor Onset and Growth Are Accelerated by p53 Reduction Among line 3 animals, the median time following initial transgene induction until a palpable tumor appeared was 10 mo, and within 16 mo, all mice developed palpable tumors (red line in [A]). In *p53^+/−^* transgenic animals (blue line in [A]), mammary tumors were detected significantly earlier (*p* < 0.0003) with a median onset of 6 mo. Among mice with BALB/cJ background (black line in [A]), median mammary tumor latency (8.5 mo) was significantly shorter (*p* = 0.0077) compared to mice of the hybrid BDF1 background strain and indistinguishable (*p* = 0.2466) from *WAP-T* *_121_* *;p53^+/−^* mice. Once palpable, *WAP-T* *_121_* *;p53^+/−^* tumors grew faster than the *p53* wild-type counterparts (B). The average growth rates for *p53^+/+^* (black solid) and *p53^+/−^* (dashed) are indicated.

All females from higher-expressing lines (lines 3 and 4) failed to nurse pups because of lactation defects and developed mammary tumors after multiple pregnancies. Because line 4 mice expressed T_121_ in nonmammary tissues, further characterization focused on line 3. For this line, the median time following initial transgene induction until a palpable tumor appeared was 10 mo, and within 16 mo, all mice developed palpable tumors (Figure 5A). Interestingly, latency in this line on a BALB/cJ background (see Materials and Methods) was reduced to a median time of 8.5 mo (*p* = 0.0077; Figure 5A) indicating the presence of modifier alleles. The condensed timeframe for tumor development in this strain will also be valuable for future preclinical studies using this model. However, all further studies in the current report were carried out on the original B6D2F1 background.

The median onset for mammary tumors in line 4 was 14 mo (*n* = 3; data not shown), which indicates that the transgene and not its insertion caused tumorigenesis. With two exceptions, line 3 *WAP-T_121 _*mice, regardless of *p53* status, developed a single palpable tumor (87% of *p53^+/+^*, *n* = 15; 78% of *p53^+/−^*, *n* = 9). A single mouse with either two or three palpable tumors was also observed in both *p53* +/+ and +/− backgrounds. At least one additional nonpalpable tumor was visible during necropsy in approximately one-third of all tumor-bearing mice. While the two lower-expressing lines, lines 1 and 2, were able to nurse pups and appeared grossly normal, both had hyperplastic lobular alveoli associated with increased levels of proliferation and apoptosis. However, females from low-expressing lines did not develop adenocarcinomas after at least four pregnancies and 20 mo of age (line 1, *n* = 2; line 2, *n* = 6) (data not shown).

**Figure 6:**
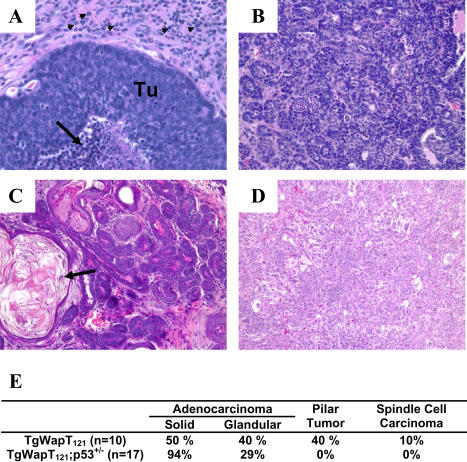
Tumor Morphologies Hemotoxylin and eosin staining of *WAP-T_121_* (C and D) and *WAP-T* *_121_* *p53^+/−^* (A and B) (also representative of *WAP-T_121_*) tumor sections shows that terminal stage adenocarcinomas have varied morphologies. Poorly differentiated solid tumors were comprised of nests (A) or cords of epithelial cells (Tu) that infiltrate a fibrous stroma and were accompanied by necrosis (arrow) and strong immune response (arrowheads). Moderately differentiated glandular tumors (B) consisted of irregular, disorganized glands. In animals of wild-type *p53* background, four pilar tumors (C), distinguished by swirls of laminar acellular keratin (arrow), and a single spindle cell carcinoma (D) were also observed. For comparison, a lactating gland from a wild-type animal is shown in Figure 3A. The percentage of animals displaying each of the phenotypes is summarized in (G). Since many tumors shared multiple morphologies, the sum exceeds 100%.

Most terminal stage tumors in either wild-type or *p53^+/−^* backgrounds were adenocarcinomas (Figure 6A, 6B, and 6E); however, we also observed four pilar tumors (Figure 6C and 6E) and one spindle cell carcinoma (Figure 6D and 6E). Terminal-stage mammary adenocarcinomas resembled poorly to moderately differentiated invasive ductal adenocarcinoma in humans. Morphologically, we designate these tumors as mixed solid and glandular carcinomas with necrosis and fibrosis. Poorly differentiated solid tumors (Figure 6A) are composed of nests of epithelial cells with large pleomorphic nuclei and delicate chromatin patterns with inverted nuclear:cytoplasmic ratios, while glandular tumors (Figure 6B) are composed of irregular glands with varying degrees of differentiation. While most animals had a single tumor mass, the adenocarcinomas were multifocal, with solid tumors consisting of subclones of distinct expansile masses, and with only two exceptions, glandular tumors were coincident with solid tumors. The adenocarcinomas were malignant, infiltrating dense, fibrous connective tissue, and were accompanied by strong peripheral immune response (Figure 6A).

### Mammary Tumor Onset and Growth Are Accelerated by p53 Reduction 

Since 75% of the apoptosis induced by Rb_f_ inactivation was mediated by *p53* and was indeed reduced even in *p53^+/−^* mice, we investigated the impact of *p53* loss on tumor onset and growth kinetics. Animals harboring either one or two *p53* null alleles were monitored for mammary tumors. As expected, a subset of *p53^+/−^* and *p53^−/−^* mice developed nonmammary tumors (either thymic lymphomas or sarcomas), consistent with published reports (Jacks et al. 1994; Sandgren et al. 1995; Dannenberg et al. 2000). All *p53^−/−^* mice (*n* = 4) succumbed to these tumors by 4 mo of age, prior to developing palpable mammary tumors, so acceleration of this phenotype could not be assessed. In *p53^+/−^* animals, mammary tumors were detected significantly earlier (see Figure 5A; *p* = 0.0003) compared with *p53^+/+^* mice. Furthermore, once palpable, *WAP-T_121_;p53^+/−^* tumors grew significantly faster than the *p53* wild-type counterparts (see Figure 5B). The observation of four pilar tumors in *p53^+/+^* animals and none in *p53^+/−^* animals is a statistically significant difference (Fisher–Freeman–Halton's exact test, *p* = 0.0177) and suggests that the reduction of p53 activity drives tumors to the adenocarcinoma phenotype. Taken together, these studies indicate that *p53* heterozygosity leads to increased tumor growth rates and/or progression and may alter the spectrum of tumor morphologies.

### Selective Pressure for p53 Inactivation during Adenocarcinoma Development

Since apoptosis was significantly reduced in *WAP-T_121_;p53^+/−^* mammary tissue compared with that of *WAP-T_121_;p53^+/+^* mice, it was possible that *p53* heterozygosity was sufficient for tumor acceleration. To assess whether this was the case or whether there was selective pressure for p53 inactivation during tumor progression, real-time PCR analysis was employed to determine the status of the wild-type *p53* allele in *WAP-T_121_;p53^+/−^* tumors. Of ten tumors, eight showed loss of the wild-type *p53* allele (Table 1), indicating that the apoptosis reduction observed in *WAP-T_121_;p53^+/−^*mammary epithelium was not sufficient for tumor progression. Significant selective pressure favored cells that had completely inactivated p53, indicating that tumor progression requires further reduction of apoptotic activity and/or that *p53* loss contributes to tumor progression through additional mechanisms that confer selective advantage. Assessment of apoptosis levels in terminal tumors showed apoptosis levels were indeed reduced in comparison to preneoplastic tissue (see Figure 4B).

**Table 1:**
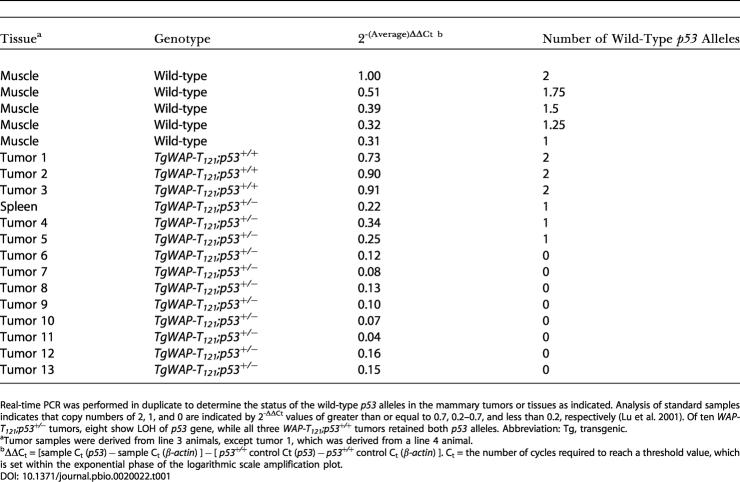
*p53* LOH among the Majority of *p53^+/−^* Tumors

Real-time PCR was performed in duplicate to determine the status of the wild-type *p53* alleles in the mammary tumors or tissues as indicated. Analysis of standard samples indicates that copy numbers of 2, 1, and 0 are indicated by 2^-ΔΔCt^ values of greater than or equal to 0.7, 0.2–0.7, and less than 0.2, respectively (Lu et al. 2001). Of ten *WAP-T_121_;p53^+/−^* tumors, eight show LOH of *p53* gene, while all three *WAP-T_121_;p53^+/+^* tumors retained both *p53* alleles. Abbreviation: Tg, transgenic

^a^Tumor samples were derived from line 3 animals, except tumor 1, which was derived from a line 4 animal

^b^ΔΔC_t_ = [sample C_t_ (*p53*) − sample C_t_ (*β-actin*) ] − [ *p53^+/+^* control Ct (*p53*) − *p53^+/+^* control C_t_ (*β-actin*) ]. C_t_ = the number of cycles required to reach a threshold value, which is set within the exponential phase of the logarithmic scale amplification plot

### Comparative Genomic Hybridization Reveals Recurrent Chromosomal Imbalances in Tumors, but Limited Chromosomal Instability

**Figure 7:**
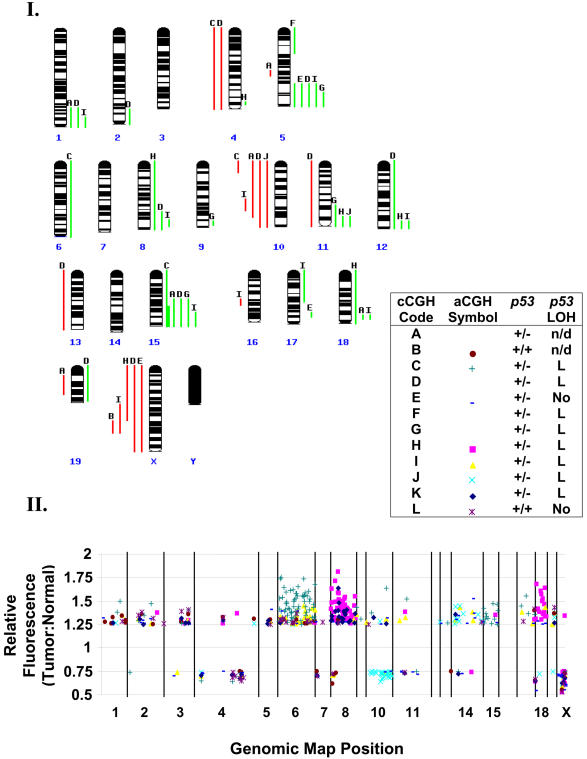
CGH Analysis Shows Limited Genomic Instability Twelve tumors were analyzed by CGH: ten by cCGH (Panel I, A–J), eight by aCGH (Panel II, B, C, E, and H–L), and six by both procedures (Panels I and II, B, C, E, and H–J). In Panel I, green and red lines adjacent to the ideograms indicate relative gain or loss, respectively. Tumor sample identities are indicated by letters above gain and loss lines. Only a single sample (Panel I, D) shows loss of Chromosome 11. Telomeric sequences of many chromosomes are increased, most frequently Chromosomes 5 and 15. Recurrent losses are seen on Chromosomes 10 and X. For aCGH (Panel II), the genomic map is depicted with chromosomes horizontally aligned centromere to telomere. The relative fluorescence intensities (tumor:normal) are indicated along the vertical axis. Individual BACs are plotted according to their physical map position versus relative fluorescence, with sample identities indicated by a unique symbol for each tumor. To simplify visualization, only BACs with relative intensities greater than 1.25 (gains) or less than 0.75 (losses) are shown. X Chromosome values were halved to account for sex-mismatched samples. Changes spanning the entire length of the chromosome are readily detected on Chromosomes 6, 8, 10, 15, 18, and X. None of the clones showing loss on Chromosome 11 spans the *p53* locus. The original *p53* background of the animal and the *p53* LOH status of each tumor are also indicated in the legend.

Among the multiple mechanisms of tumor suppression attributed to p53, a common hypothesis is that p53 prevents genetic instability. Indeed, studies using other mouse models indicate loss of p53 function in tumors often correlates with chromosomal instability. These include other breast cancer models such as *Wnt-1p53^+/−^* (Donehower et al. 1995) and *MMTV-ras p53^+/−^* (Hundley et al. 1997) and *p53^+/−^* thymic lymphomas and sarcomas (Venkatachalam et al. 1998). In marked contrast, our study of p53 deficiency in an evolving brain epithelial tumor showed that tumorigenesis progresses without chromosomal instability, indicating p53 loss contributes via alternative mechanisms (Lu et al. 2001). To determine whether this difference was due to cell-type specificity, differences in initiating mechanisms, or differences in experimental approaches, we analyzed the genome of mammary *WAP-T_121_;p53^+/−^* tumors. We employed two methods of comparative genomic hybridization (CGH): chromosome-based CGH (cCGH) (Panel I in Figure 7) (Kallioniemi et al. 1992) and microarray CGH (aCGH) (Panel II in Figure 7) (Solinas-Toldo et al. 1997; Pinkel et al. 1998).

Twelve mammary tumors were assayed by CGH: ten by cCGH, eight by aCGH, and six by both procedures. Both assays revealed limited genomic imbalances (Figure 7), yet only a single tumor showed loss of Chromosome 11 (which harbors *p53*). Among samples tested by both methods, there is strong concordance among large chromosomal changes, encompassing multiple cytological bands to whole chromosome lengths. For example, there is apparent whole chromosomal duplication of Chromosomes 6 and 15 in tumor C and of Chromosomes 8 and 18 in tumor H, monosomy of Chromosome 10 in tumor J, and loss of X Chromosome in tumors E and H (all or partial, respectively). Making comparisons among imbalances spanning shorter chromosome lengths was more difficult, mainly due to the challenge of reconciling cytological and physical maps. Furthermore, technical limitations may account for real differences between the two assays: small imbalances detected by one to several bacterial artificial chromosome (BAC) clones are irresolvable by cCGH; on the other hand, the relatively low density of BAC clones may not adequately sample smaller regions detected by cCGH. Nevertheless, on average, about five imbalances per tumor were detected by cCGH. This number is comparable to the number of changes observed in myelocytomatosis oncogene (c*-myc*)-induced mouse mammary tumors (Weaver et al. 1999) and human tumors (Ried et al. 1995), yet less than the number of changes seen in breast cancer 1 (*Brca1*)-deficient mouse tumors (8.0) and more than v-*erb-b2* erythroblastic leukemia viral oncogene homolog 2 (HER2/*neu*)-induced tumors (2.8) (Montagna et al. 2002; Weaver et al. 2002).

## Discussion

### Common Mechanisms for Tumor Progression in Epithelial Cells of Distinct Origin

Here we report that loss of pRb family function in mammary epithelium predisposes to malignant adenocarcinoma. Using a single transgenic allele, we have thus far inactivated the pRb pathway in several cell types in the mouse: brain choroid plexus epithelium, astrocytes, and mammary epithelium. In each case, despite the marked differences among these divergent cell types, pRb inactivation causes a similar response, initially evoking increased proliferation and apoptosis and, ultimately, predisposing to tumorigenesis (Chen et al. 1992; Saenz-Robles et al. 1994; Symonds et al. 1994; Xiao et al. 2002).

Not surprisingly, the long latency of mammary adenocarcinomas indicates that additional events are required for tumor progression. We show that mammary epithelium is similar to brain epithelium (Symonds et al. 1994; Lu et al. 2001) in its requirement for p53 activity in the apoptotic response to aberrant proliferation caused by pRb_f_ inactivation. Previous models using wild-type large T antigen (Li et al. 1996b; Husler et al. 1998; Green et al. 2000; Schulze-Garg et al. 2000) are unable to address the relative contribution of pRb and p53, since T antigen also binds and inactivates p53. As in brain epithelium, we show here that when the mammary tumor phenotype is initiated by pRb_f_ inactivation, most of the apoptosis is mediated through p53. Furthermore, as in brain epithelium, heterozygosity for a null *p53* allele significantly shortens tumor latency (discussed further below). Importantly, the Rb_f_ deficiency-induced apoptotic response and inhibition of tumor progression are not universally dependent on p53. In astrocytes, we recently showed that PTEN, and *not* p53, modulates these same responses to Rb_f_ inactivation. In contrast to the p53-dependent apoptosis of mammary epithelial cells in response to pRb_f_ deficiency, apoptosis associated with normal mammary involution subsequent to lactation does not require p53 (Li et al. 1996a). Thus, the “wiring” of the apoptotic response within this cell type is not global, but rather depends on the signal.

Although loss of p53-dependent apoptosis accounts for the acceleration of mammary tumorigenesis in *WAP-T_121_;p53^+/−^* mice, in models expressing either activated *v-Ha-ras* (Hundley et al. 1997) or *Wnt-1* (Jones et al. 1997), earlier tumor formation in p53 heterozygous and homozygous null mice is accounted for by increased proliferation rather than attenuated apoptosis. An important caveat to this comparison is that the latter studies compared apoptosis in terminal tumors in which loss of apoptosis might have been selected regardless of initial p53 status, leaving open the possibility that tumor growth rates in these models reflect the combined effects of increased proliferation as well as reduced apoptosis. Nevertheless, there is a clear difference in *WAP-T_121_* mammary gland in that, unlike the *Ras* and *Wnt-1* models, proliferation levels do not depend on *p53* status. Taken together, these observations indicate that the specific cellular response to an oncogenic stimulus depends on the nature of the initial insult. Given that the pRb pathway is directly disrupted in T_121_-expressing cells, this could be explained if these other initiating events evoke p53-dependent growth arrest which, in part, functions upstream of pRb.

### High Selective Pressure for p53 Inactivation in the Transition to Aggressive Mammary Adenocarcinoma

Most of the apoptosis induced by pRb_f_ deficiency in both mammary (75%) and brain (85%) epithelia is *p53*-dependent as determined by comparing *p53^+/+^* and *p53^−/−^* tissue. However, while *p53* heterozygosity had no impact on the level of apoptosis in the brain epithelium, in the mammary gland the level was reduced by half in *p53^+/−^* tissue. Given that apoptosis is the basis for selective inactivation of p53 in the brain tumor model (Lu et al. 2001; X. Lu and T. Van Dyke, unpublished data), it was possible that the pressure was relieved or reduced in *WAP-T_121_;p53^+/−^* mice. However, aggressive adenocarcinoma growth was accelerated with 100% penetrance, and 80% of these tumors underwent selective loss of the wild-type *p53* allele, just as in the brain tumor model (Lu et al. 2001). This result indicates that tumor progression requires more than a simple reduction in the level of apoptosis; it follows that *p53* may contribute to tumor suppression by multiple mechanisms.

While both mammary and brain carcinomas show high rates of *p53* loss of heterozygosity (LOH), the mechanism of loss may be distinct. Chromosome loss clearly explains *p53* LOH in the brain carcinoma model (Lu et al. 2001) where nearly all tumors (greater than 90%) are monosomic for Chromosome 11, whereas only a single mammary tumor analyzed by CGH showed Chromosome 11 loss. Alternative mechanisms that may explain *p53* LOH in the mammary tumors include somatic recombination or chromosomal reduplication following mitotic nondisjunction. Whether these alternative routes of LOH represent bona fide tissue-specific phenomena or are due to relatively small sample sizes will require further analyses. Interestingly, most mammary tumors derived from *Brca1*-deficient mice lost *p53*; however, regions distal to *p53* were amplified (Weaver et al. 2002). Thus, it is possible that mammary tumor promoting factor(s) is located on distal Chromosome 11, selecting *against* loss.

### Limited Chromosomal Instability in the Absence of p53

Genomic instability is a hallmark of most human solid tumors, and a widely held view is that p53 represses instability to suppress tumorigenesis, although evidence for this activity has been mostly correlative. Contrary to this model, we demonstrated previously that in the absence of p53 activity in brain epithelia, tumors progress without chromosomal instability; except for Chromosome 11 loss, in a *p53^+/−^* background these carcinomas are diploid (Lu et al. 2001). Here we show that mammary tumors similarly harbor limited genome-wide alterations. While the number of aberrations within the mammary tumors is small, it is intriguing that some changes are recurrent, suggesting that their accrual is causal in tumorigenesis. T_121_-induced mammary carcinomas harbor more genomic imbalances than brain tumors (approximately five versus approximately one). One explanation for this observation is that, because the brain is a vital organ, animals succumb to their illness when the brain tumor is at a relatively earlier stage at which fewer changes have accumulated. However, chromosome content of choroid plexus tumors passaged further in xenografts remained stable (X. Lu and T. Van Dyke, unpublished data). The converse experiment, analyses of early mammary tumors subsequent to p53 loss, will be required to determine the kinetics of chromosomal changes in this tissue.

### Pocket Protein Redundancy

Chimera and tissue-grafting experiments with pRb-deficient cells indicate the absence of pRb alone is not sufficient for abnormal mammary development or tumor formation (Maandag et al. 1994; Robinson et al. 2001). Yet mammary-directed overexpression of CCND1, an upstream regulator of pRb_f_, leads to mammary adenocarcinoma (Wang et al. 1994). Given other recent studies indicating the possibility for compensation of pRb function by p107 and/or p130 (Dannenberg et al. 2000; Sage et al. 2000) and the clear redundancy of function in some murine cell types (Robanus-Maandag et al. 1998; Xiao et al. 2002), it is likely that the discrepancy among our results can be explained by overlapping functions of other family members, p107 and/or p130. In our studies, T_121_ abrogates the activities of all *Rb* family members by a dominant interfering mechanism. A subtly distinct alternative explanation is that the acute loss of pRb signaling, rather than a chronic loss as of pRb during mammary development, as in the chimera and grafting models, accounts for the difference. Cell culture experiments that support this hypothesis were recently reported (Sage et al. 2003). In this model, p107 and p130 may be more responsive to pRb regulatory signals during development than in the terminally differentiated tissue; therefore, the developing tissue more easily accommodates for the absence of pRb in the pool of available pocket proteins. In the *WAP-T_121_* model, the gland undergoes normal development and then is subsequently subjected to acute pRb pathway loss. We presume that this scenario more closely mimics the situation of spontaneous somatic loss in adult human breast. The test of this alternative hypothesis awaits analyses of tissue-specific inactivation of pRb and the paralogous pocket proteins using conditional alleles.

### A Model for Mammary Tumorigenesis Initiated by Targeting the pRb Pathway

The *WAP-T_121_* model is a significant addition to the current repertoire of preclinical mammary tumor models exploring the role of pRb pathway in tumorigenesis. Despite the prevalence of pRb pathway defects in human sporadic cancers, mice harboring germline mutations of *p16^INK4a^* do not develop mammary cancer (Krimpenfort et al. 2001; Sharpless et al. 2001). In addition, mammary-directed expression of CCND1 is only mildly oncogenic (Wang et al. 1994), and as mentioned above, inactivation of pRb alone is not sufficient for tumorigenesis. Although the WAP promoter was a convenient means of directing mammary-specific expression for an initial assessment this model, it also presents the major shortcoming to this model in that expression of T_121 _is linked to lactogenic hormone activity, as in most existing murine mammary tumor models. Future improvements aim to direct expression of T_121_ through hormone-independent methods. Finally, the advantage over wild-type T antigen models is that *WAP-T_121_* uncouples the simultaneous inactivation of pRb and p53 and permits an assessment of the relative contributions of the individual oncogenic pathways. Testing the combinatorial effects of *Rb* loss and other breast cancer mutations (e.g., *BRCA1* and *BRCA2*), along with the further characterization of *WAP-T_121_* tumors, should help provide additional insights into human breast cancer biology.

## Materials and Methods

### 

#### Derivation and characterization of transgenic mice.

The 2.4 kb WAP promoter region was isolated from a WAP-TGFα construct (a gift from David Lee, University of North Carolina at Chapel Hill, United States [Sandgren et al. 1995]) and was cloned upstream of a 2.4 kb KpnI–SalI fragment of the *dl*1137′t plasmid (Chen et al. 1992). We targeted T_121_ expression to mammary gland using the WAP promoter, which is induced late in pregnancy and expressed during lactation (Pittius et al. 1988) (see Figure 1). T_121_ contains the first 121 amino acids of the SV40 T antigen (see Figure 1) that encodes a J domain and a pRb-binding domain, which together are sufficient to cause transformation by inactivating the pRb_f_ proteins (DeCaprio et al. 1989; Dyson et al. 1989; Ewen et al. 1989). Importantly, in contrast to other wild-type T antigen constructs encoding the entire SV40 early region (Husler et al. 1998; Green et al. 2000; Schulze-Garg et al. 2000), small t antigen expression is absent due to a deletion that removes the splice acceptor site. The importance of this is demonstrated by the recent observation that small t antigen alone is sufficient for tumorigenesis in the mammary gland (Goetz et al. 2001). Furthermore, p53 and EP300 (E1A-binding protein p300), which map to the carboxyl half of T antigen, are also abolished, thus permitting assessment of pRb_f_ inactivation without the confounding effects of altering additional suppressor pathways. An EcoRI fragment containing the full transgene (see Figure 1) at a concentration of 4 ng/μl was injected in to fertilized eggs harvested from B6D2F1 (Jackson Laboratory, Bar Harbor, Maine, United States) mice as described previously (Yan et al. 1990). Transgenic mice were identified by PCR amplification of a 160 bp fragment using primers 5′-GAATCTTTGCAGCTAATGGACC-3′ and 5′-GCATCCCAGAAGCTCCAAAG-3′ with toe-derived genomic DNA as template. Cycling profile was as follows: 94°C, 2 min; 35 cycles of 94°C, 20 s; 62°C, 45 s; 72°C, 45 s; and final incubation of 72°C, 2 min. *TgWAP-T_121_* mouse lines were maintained by crossing to nontransgenic B6D2F1 mice (Jackson Laboratory) and therefore are designated as B6;D2-Tg(WAP-T_121_) Tvd. To study the effect of background differences, *WAP-T_121_* males were backcrossed to BALB/cJ (Jackson Laboratory) female mice. To increase sample size, tumor onset analysis for BALB/c background mice combined data for N6 (*n* = 6), N7 (*n* = 1), and N9 (*n* = 4) generation mice. For tumor induction, female mice, unless noted as virgin, were housed with male mice to maximize the number of pregnancies, because WAP promoter activity is lactation-dependent (Pittius et al. 1988).

To study the effect of *p53* mutation on mammary tumorigenesis in *WAP-T_121_* mice, male *WAP-T_121^+^_* mice were mated to *p53^+/−^* females (*p53^tm1Tyj^*; Jackson Laboratory). *p53* genotypes were determined by PCR using two reactions (Lowe et al. 1993), one that amplifies the neomycin insertion site (neomycin primer: 5′- TCCTCGTGCTTTACGGTATC-3′, *p53* primer: 5′-TATACTCAGAGCCGGCCT-3′; 525 bp product) and a second that amplifies the endogenous *p53* allele (substituting 5′-ACAGCGTGGTGGTACCTTAT-3′ for the *neo* primer, 475 bp product). Cycling parameters were the same as the above *WAP-T_121_* reaction. We performed the cross *WAP-T_121^−^_;p53^+/−^* × *WAP-T_121^+^_;p53^+/−^,* and transgenic female mice that were *p53^+/+^*, *p53^+/−^*, or *p53^−/−^* were used for analyses while nontransgenic littermates served as controls.

#### Western immunoblotting analysis.

Protein expression levels were assayed as previously described (Symonds et al. 1993). Fresh or flash-frozen tissue samples were homogenized in lysis buffer (50 mM Tris [pH 8.0], 5 mM EDTA, 150 mM NaCl , and 1% NP-40) using a Polytron^®^ homogenizer (Kinematica, Littau-Lucerne, Switzerland). Total protein (10 μg) was electrophoresed through a 15% polyacrylamide denaturing gel and then transferred to nitrocellulose membrane (15 V, 30 min). Alternatively, for low-expressing lines, immunoprecipitation was performed prior to electrophoresis as previously described (Symonds et al. 1991). The filter was preincubated in 3% bovine serum albumin, followed by incubation with primary antibody against SV40 T antigen (PAb419 at a dilution of 1:5,000; Harlow et al. 1981). The filter was then washed, followed by incubation at room temperature with horseradish peroxidase-conjugated goat anti-mouse IgG (Amersham Biosciences, Little Chalfont, United Kingdom). The enhanced chemiluminescence method (Amersham Biosciences) was used for autoradiography.

#### Histopathology and immunohistochemistry.

Mammary tissue and tumor samples were dissected from *WAP-T_121_* transgenic or age- and parity-matched B6D2F1 animals. A portion of each tumor was flash-frozen in liquid nitrogen and the remaining tissue was fixed in 10% phosphate buffered formalin, embedded in paraffin, cut to a 5-μm thickness, and stained with hemotoxylin and eosin or immunostained using the Vector ABC system (Vector Laboratories, Burlingame, California, United States) for histopathological examination. Apoptosis levels were evaluated by TUNEL assay (Gavrieli et al. 1992) essentially as described in Symonds et al. (1994).

#### Real-time PCR.

Quantitative real-time PCR analysis was performed using a TaqMan approach on DNA derived from terminal tumors to determine the status of the wild-type *p53* allele as previously described (Lu et al. 2001). The primers for the *p53* allele were 5′-ATGGCCATCTACAAGAAGTCACAG-3′ and 5′-ATCGGAGCAGCGCTCATG-3′. The sequence of the *p53* probe was 5′-ACATGACGGAGGTCGTGAGACGCTG-3′. The primers for the internal control *β-actin* gene were 5′-AAGAGCTATGAGCTGCCTGA-3′ and 5′-ACGGATGTCAACGTCACACT-3′. The sequence of the *β-actin* probe was 5′-CACTATTGGCAACGAGCGGTTCCG-3′. Each 25-μl reaction mixture contained 50 ng of DNA template, 18 nM *p53* primers, 80 nM *β-actin* primers, 8 nM probe, and 12.5 μl of TaqMan Universal PCR Master Mix (Applied Biosystems, Foster City, California, United States) containing AmpliTaq Gold polymerase, deoxynucleoside triphosphates, and PCR buffer. The cycling conditions were 50°C for 2 min and 95°C for 10 min for 1 cycle, and 95°C for 15 s and 60°C for 1 min for 40 cycles. The reactions were performed using an ABI 7700 Sequence Detection system (Applied Biosystems), and the data analyzed using Sequence Detector 1.7 (Applied Biosystems) and standard protocols (http://www.appliedbiosystems.com). The copy number of each sample was determined by calculating ΔΔCt based on the formula ΔΔC_t_ = [sample C_t(p53)_ − sample C_t(_ *_β-actin_*_)_] − [p53^+/+^ control Ct_(p53)_ − p53^+/+^ control Ct_(_ *_β-actin_*_)_], where C_t_ is the number of cycles required to reach a threshold based on linear amplification. Analyses of standard samples (L. Chin, Harvard University, Cambridge, Massachusetts, United States, personal communication) indicate copy numbers of 2, 1, and 0 are indicated by 2^-ΔCtn^ values of greater than 0.6, 0.15–0.6, and less than 0.15, respectively. Standard samples analyzed along with experimental samples confirmed the accuracy of these assignments.

#### Statistical analyses.

Kaplan–Meier survival analysis was used to determine median tumor latencies (StatsDirect, Camcode, Sale, United Kingdom), and the Log-Rank (Peto, StatsDirect) test was performed to evaluate significance. The equivalence of tumor morphology distributions was tested using the Fisher–Freeman–Halton's exact test.

#### CGH.

Genomic DNA was extracted from end-stage tumors (1 cm in diameter) or tails using a DNeasy genomic tip (Qiagen, Valencia, California, United States) and further purified by proteinase K digestion followed by phenol/chloroform extraction, ethanol precipitation, and resuspension in sterile H_2_O. cCGH was performed as described in Kallioniemi et al. (1992), Donehower et al. (1995), and Lu et al. (2001). aCGH was performed as described in Snijders et al. (2001). For both methods, genomic DNA from tumor and normal tissue was labeled with different fluorochromes and then cohybridized together with *Cot-1* DNA to either normal metaphase chromosomes from cultured cells (cCGH) or microarrayed BAC clones containing mouse genomic DNA (aCGH). Nonequivalent fluorescence intensities indicate relative imbalances of genomic DNA. Aneuploidy and partial chromosome gains and losses are detectable by cCGH with approximately 10 Mb resolution. Graphical output of cCGH data was generated using the National Cancer Institute and National Center for Biotechnology Information Spectral Karyotyping *SKY* and Comparative Genomic Hybridization CGH Database (http://www.ncbi.nlm.nih.gov/sky/skyweb.cgi). For aCGH, approximately 1,500 BAC clones span the entire mouse genome with 2–20 Mb spacing. Tumor DNA and normal DNA were sex-mismatched; thus, the X Chromosome served as an internal control, while normal tail DNA was used as a negative control. Gains or losses were scored based on tumor:normal fluorescence ratios that were greater than 1.25 or less than 0.75, respectively.

## Supporting Information

### 

#### Accession Numbers

The accession numbers for the genes and gene products discussed in this paper are *Brca1* (LocusLink ID 12189), CDK2 (LocusLink ID 1017), CDK4 (LocusLink ID 1019), CDK6 (LocusLink ID 1021), c*-myc* (LocusLink ID 17869), cyclin D1 (LocusLink ID 595), cyclin E (LocusLink ID 898), E2F (InterPro ID IPR003316), HER2/*neu* (LocusLink ID 13866), histone deacetylase (LocusLink ID 3065), p16^INK4a^ (LocusLink ID 1029), p53 (LocusLink ID 7157), p107 (LocusLink ID 5933), p130 (LocusLink ID 5934), p300 (LocusLink ID 2033), PCNA (LocusLink ID 18538), pRb (LocusLink ID 5925), *PTEN* (LocusLink ID 5728), v*-Ha-ras* (LocusLink ID 3265), WAP (LocusLink ID 22373), and *Wnt-1* (LocusLink ID 22408).

These databases may be found at www.ncbi.nlm.nih.gov/LocusLink/ (LocusLink), and www.ebi.ac.uk/InterPro/ (InterPro).

## Abbreviations

aCGH: microarray comparative genomic hybridization
BAC: bacterial artificial chromosome
Brca1: breast cancer 1
cCGH: chromosome comparative genomic hybridization
CCND1: cyclin D1
CCNE1: cyclin E1
CDK2: cyclin-dependent kinase 2
CDK4: cyclin-dependent kinase 4
CDK6: cyclin-dependent kinase 6
CGH: comparative genomic hybridization
c*-myc*: myelocytomatosis oncogene
LOH: loss of heterozygosity
MMTV: murine mammary tumor virus
p16^INK4a^: cyclin-dependent kinase inhibitor 2A
PCNA: proliferating cell nuclear antigen
pRb: retinoblastoma 1
pRb_f_: retinoblastoma protein family
PTEN: phosphatase and tensin homolog
SV40: simian virus 40
v-*erb-b2*: erythroblastic leukemia viral oncogene homolog 2
v*-Ha-ras*: Harvey rat sarcoma viral oncogene
WAP: whey acidic protein
Wnt-1: *wingless*-related MMTV integration site 1
